# Genomic Functional Analysis and Cellulase Characterization for the Enzyme-Producing Strain *Bacillus subtilis* Y4X3 Isolated from Saline–Alkaline Soil in Xinjiang, China

**DOI:** 10.3390/microorganisms13030552

**Published:** 2025-02-28

**Authors:** Xinrun Yao, Min Lin, Yongliang Yan, Shijie Jiang, Yuhua Zhan, Bodan Su, Zhengfu Zhou, Jin Wang

**Affiliations:** 1 College of Life Science and Engineering, Southwest University of Science and Technology, Mianyang 621000, China; yaoxinrun@mails.swust.edu.cn (X.Y.); linmin@caas.cn (M.L.); sjjiang0406@swust.edu.cn (S.J.); 2 National Key Laboratory of Agricultural Microbiology, Biotechnology Research Institute, Chinese Academy of Agricultural Sciences, Beijing 100081, China; yanyongliang@caas.cn (Y.Y.); zhanyuhua@caas.cn (Y.Z.); subodan@caas.cn (B.S.); 3 Key Laboratory of Agricultural Microbiome (MARA), Biotechnology Research Institute, Chinese Academy of Agricultural Sciences, Beijing 100081, China

**Keywords:** *Bacillus subtilis* Y4X3, saline–alkaline soil, genomic function, alkali-resistant cellulase, Cel5A

## Abstract

Biotechnological research and application of microbial enzyme production have consistently been focal points for scientific inquiry and industrial advancement. In this study, *Bacillus subtilis* Y4X3 was isolated from saline–alkaline soil in Xinjiang, China. Extracellular enzyme production analysis revealed that *B. subtilis* Y4X3 can secrete various enzymes, including cellulase, xylanase, protease, and amylase. Sequencing and assembly of the complete genome of this strain revealed a genome size of 4,215,636 bp with 43.51% C + G content, including 4438 coding genes. Genome annotation was performed with databases to predict gene functions in *B. subtilis* Y4X3, and a variety of genes related to carbohydrate metabolism were identified. A cellulase-encoding gene was subsequently cloned from the genome and heterologously expressed in *Escherichia coli*. The optimum pH and temperature for the purified cellulase Cel5A were 5.0 and 60 °C, respectively. Stability analysis revealed that Cel5A remained stable at pH 5.0–9.0 and 20–60 °C; after 1 h at pH 9.0, the relative enzyme activity still exceeded 60%. Additionally, Cel5A was positively affected by various metal ions and exhibited good tolerance to multiple chemical reagents. The results indicate that *B. subtilis* Y4X3 has the potential to produce a variety of industrial enzymes and could serve as a promising candidate for more efficient and cost-effective industrial applications; the characterized thermostable and alkali-resistant cellulase Cel5A also has potential applications in biotechnology and industry.

## 1. Introduction

Investigation of microbial enzymes and their industrial and biotechnological applications remains pivotal for advancing scientific and industrial endeavors [1,2]. In recent decades, enzymes derived from microorganisms, including cellulases, lipases, proteases, amylases, and chitinases, have been extensively used in diverse industries, such as detergents, textiles, food processing, animal feed, pharmaceutical industries, bioremediation, and bioenergy production [3]. *Bacillus subtilis*, a Gram-positive bacterium, is the most extensively studied species within the genus *Bacillus* and can be isolated from diverse environments [4]. Its efficient protein secretion system and adaptable metabolism, especially its great potential in enzyme production, have led to its widespread application [5]. For example, *Bacillus* species are major producers of proteases, accounting for approximately 60% of global enzyme sales, which are utilized in the detergent industry for stain removal and in food processing [6]. Additionally, the heat-stable, alkali-resistant endopolysaccharide enzyme produced by *B. subtilis* DFR40 has applications in the synthesis of prebiotic xylooligosaccharides [7]. Functional genomic analysis of *Bacillus* is crucial for elucidating microbial biological properties, enhancing the production efficiency of enzymes for industrial applications, and developing novel active metabolites [8].

Cellulases are highly active biocatalysts widely utilized in the conversion and utilization of cellulose [9]. They constitute a class of complex enzyme systems that can degrade cellulose molecules to oligosaccharides or glucose and belong to the glycoside hydrolases (GHs) family [10]. Cellulases are categorized into three primary types on the basis of their site of action and released products: endo-1,4-β-D-glucanase (EC 3.2.1.4), exo-1,4-β-D-glucanase (EC 3.2.1.91), and β-glucosidase (EC 3.2.1.21) [11]. According to the statistics, cellulases rank third in terms of sales in the global enzyme market [12], with cellulase sales reaching $8.95 billion in 2023. The importance of these enzymes is increasingly being recognized in various fields, such as the food and beverage industry, the detergent and textile industry, animal feed production, and biofuel production. Cellulases help in the clarification of fruit and vegetable juices [13], biopolishing of cotton fabrics [14], and conversion of cellulose to fermentable sugars for the production of biofuels [15], which is important for realizing a green circular economy. Currently, commercial cellulases are derived predominantly from fungi, particularly *Trichoderma reesei*, and relatively few are sourced from bacteria, particularly those of the genus *Bacillus* [16]. The natural cellulase Cel5A [17] from *T. reesei* exhibited optimal activity at pH 4.6 to 5.0, with decreased activity observed above pH 7.0, making it suitable for application under only acidic conditions. A variety of commercial enzymes from Aspergillus Ricci have the same characteristics. The cellulase Cel9z [18] from *Bacillus licheniformis* strain Z9 has high enzyme activity at pH 5.5 to 8.0, with an optimal temperature of approximately 30 °C and a significant decrease in enzyme activity observed above 60 °C; thus, the thermal stability of this enzyme needs to be improved. Further optimization of pH or temperature stability is required for a variety of cellulases to attain the conditions of industrial production [19,20,21,22].

This study focused on *B. subtilis* Y4X3, a strain isolated from soil in Xinjiang, China, with the aim of revealing its enzyme production potential through genomic functional analysis and characterizing its cellulase. Genome functional analysis of this strain provided insights into the mechanisms of carbohydrate degradation, as well as its potential value in industrial applications. By thoroughly investigating the properties of Cel5A, we aimed to contribute new enzyme resources for industrial biotechnology and establish a scientific foundation for the development of microbial resources in Xinjiang.

## 2. Materials and Methods

### 2.1. Strain Isolation and Culture

Microorganisms were isolated from saline–alkaline soil samples collected in Xinjiang, China (N42°19′78″, E86°66′52″). Ten grams of soil was suspended in 90 mL of sterile water and shaken at 180 rpm at 28 °C for 30 min. The suspensions were diluted 10^−2^, 10^−3^, and 10^−4^ with PBS, and 200 μL of each suspension was spread on LB and R2A agar media and incubated at 28 °C until single colonies had grown. After multiple rounds of streak purification, single colonies were selected for 16S rRNA sequencing and preserved as individual strains.

### 2.2. Genomic DNA Extraction and Sequencing

Total bacterial DNA was extracted for whole-genome sequencing following the protocol for the Bacterial Genomic DNA Isolation Kit (Mei5 Biotechnology Co., Ltd., Beijing, China). After the splices, short fragments, and low-quality data were filtered out, genomic assembly was performed using reads obtained from third-generation Nanopore sequencing and second-generation Illumina sequencing, with Unicycler software (Version: 0.5.0) [23] employed for the assembly process.

### 2.3. Phylogenetic Tree Construction

The 16S rDNA gene of the strain was amplified using the bacterial universal primers 27F (5′-AGAGTTTGATCCTGGCTCAG-3′) and 1492R (5′-GGTTACCTTGTTACGACTT-3′), yielding a fragment of approximately 1500 bp. Sequence comparisons were performed via the NCBI BLAST (Version: 2.10.1) tool, and strains with high homology in the NCBI database were selected for phylogenetic tree analysis. The phylogenetic tree was constructed using MEGA 11 software (Version: 11.0.13) via the neighbor-joining (NJ) method [24]. Strain identity was further confirmed through multilocus sequence typing (MLST) using multiple housekeeping genes [25].

### 2.4. Identification of Extracellular Enzyme Production

One hundred microliters of *B. subtilis* Y4X3 bacteriophage protected with glycerol stock stored at −80 °C was inoculated into 5 mL of liquid LB medium, followed by overnight incubation at 30 °C and shaking at 220 rpm. Subsequently, 10 μL of the bacterial culture was spotted onto media for the identification of cellulase, xylanase, protease, and amylase activities. After incubation for 1–3 days at 30 °C, the cultures were either directly observed or stained to assess enzyme production. The presence of enzyme activity was determined by observing the formation of degradation zones.

### 2.5. Functional Annotation of Genomes

Coding genes were predicted from the assembled genome using Prokka (version: 1.14.6) [26]. In order to obtain comprehensive gene function information, we annotated the genes against eight major databases, including the UniProt [27], KEGG [28], KEGG Pathway, GO [29], Pfam, COG [30], TIGRFams, RefSeq, and NR databases. The predicted gene sequences were compared with functional databases such as COG, KEGG, UniProt, and RefSeq using BLAST+ (version 2.11.0+) [31] to derive gene function annotations. The CAZy database, which specializes in the genomic, structural, and biochemical analysis of carbohydrates, was used for protein sequence annotation with HMMER [32]. Additionally, the signal peptides and transmembrane structural domains of the protein sequences were predicted by SignalP 6.0 and TMHMM 2.0, respectively.

### 2.6. Heterologous Expression of the Cellulase Gene in Escherichia coli

Since the *B. subtilis* Y4X3 strain exhibited robust cellulose degradation capabilities in extracellular enzyme production analysis, a cellulase gene was subsequently identified in its whole-genome sequence. Using the strain’s genomic DNA as the template, the cellulase gene without the signal peptide coding sequence was amplified by PCR and subsequently cloned and inserted into the pET28a (+) vector, which contains a C-terminal His-tag. The primers used were F (5′-AGAAGGAGATATACCATGGGGCGCAGAGACAAAAAC-3′) and R (5′-TGGTGGGTGGTGGTGCTCGAGATTGGGGTTCTG-3′), incorporating Nco I and Xho I restriction sites, respectively. The recombinant vector was initially transformed into *Escherichia coli* DH5α competent cells, confirmed by colony PCR and sequencing, and then transformed into *E. coli* BL21 (DE3) cells using heat shock [33]. Protein expression was induced with 0.4 mM isopropyl β-D-thiogalactopyranoside (IPTG), followed by cell growth at 16 °C and 200 rpm. The cellulase was purified by nickel-nitrilotriacetic acid (Ni-NTA) affinity chromatography and eluted with gradient increments of imidazole buffer. The target protein was detected by sodium dodecyl sulfate-polyacrylamide gel electrophoresis (SDS-PAGE).

### 2.7. Biochemical Characterization of Cellulases

#### 2.7.1. Effects of pH and Temperature on Cellulase

Cellulase activity was quantified by measuring the amount of reducing sugars released by the enzyme using the 3,5-dinitrosalicylic acid (DNS) method with 1% CMC-Na as the reaction substrate [34,35]. Protein concentration was determined using the BCA Protein Quantification Kit (Novozymes Vazyme Biotech Co., Ltd., Beijing, China). 20 μL of purified enzyme was mixed with 80 μL of buffer added to 100 μL of substrate and reacted at 50 °C for 30 min. Enzyme assays were conducted in triplicate to ensure data consistency and reliability. One unit of cellulase activity (U) is defined as the amount of enzyme required to liberate 1 μmol of reducing sugar per minute under the assay conditions. Specific activity is expressed as U/mg. The commercial enzyme Celluclean^®^ 5000 L (Novozymes, Bagsværd, Denmark) was employed as a positive control, while an inactivated enzyme solution served as the blank control.

The optimal pH for cellulase activity was determined using a series of buffers: citric acid-sodium citrate solution (pH 3.0–6.0), disodium hydrogen phosphate-sodium dihydrogen phosphate solution (pH 7.0–8.0), and glycine-sodium hydroxide solution (pH 9.0–12.0). The reaction was carried out at 50 °C for 30 min, and enzyme viability was determined in buffers of different pH values. The optimal temperature was then evaluated by conducting the assay at temperatures ranging from 20 °C to 100 °C under the optimal pH conditions. The effect of pH on cellulase stability was assessed by incubating the enzyme in buffers ranging from pH 3 to 12 at 50 °C for 1 h, followed by a residual enzyme activity assay. The effect of temperature on cellulase stability was determined by incubating the enzyme at different temperatures for 1 h followed by residual enzyme activity determination under the optimum conditions [36]. The highest enzyme activity was measured using the untreated enzyme solution and was set as 100%, and the relative enzyme activity under other conditions was calculated accordingly.

#### 2.7.2. Substrate Specificity of Cellulase

To assess the substrate specificity of the cellulase [37], solutions of sodium carboxymethyl cellulose (CMC-Na), crystalline cellulose (Avicel), beechwood xylan, β-mannan, sucrose, and laminarin were prepared at a final concentration of 1% and used as hydrolysis substrates. The enzyme solution was mixed with each substrate, and the enzymatic reaction was conducted at the optimal pH and temperature for 30 min.

#### 2.7.3. Effects of Metal Ions and Chemical Reagents on Cellulase

To evaluate the tolerance of cellulase to various metal ions and chemical reagents [38,39], the enzyme solution was preincubated with different reagents at 50 °C for 30 min. Subsequently, the enzymatic reaction was performed under optimal conditions. The enzyme activity in the absence of any additives was set as 100%.

#### 2.7.4. Kinetic Parameters of Cellulase

Enzymatic reactions were conducted using CMC-Na at various concentrations as the substrate, which was incubated with purified cellulase under optimal conditions for 30 min. The kinetic parameters (Km and Vmax) were determined by fitting the data to the Michaelis–Menten equation. Data analysis was performed using Origin 2021 software.

## 3. Results

### 3.1. Phylogenetic Analysis

**Figure 1:**
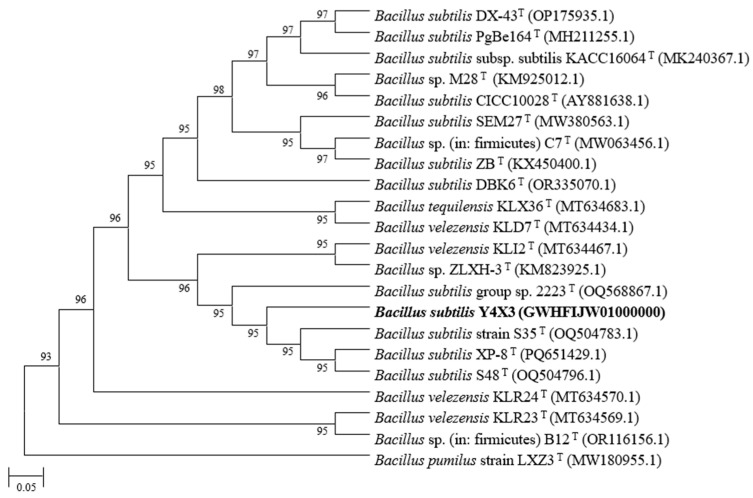
Phylogenetic tree of the 16S rRNA gene sequence generated by the neighbor-joining (NJ) method. The numbers at nodes indicate the levels of bootstrap support (>70%) based on 1000 resamplings. Bold represents the strains in this study. ‘T’ for type strain.

To elucidate the taxonomic position of the isolated strains, their 16S rDNAs were analyzed in comparison with those of closely related strains in the NCBI database, and a phylogenetic tree was constructed (Figure 1). In the comparative analysis, the 16S rDNA of *B. subtilis* Y4X3 showed over 99% similarity with those of *B. subtilis* group sp. 2223, *B. subtilis* S35, *B. velezensis* KL12, *B. subtilis* S48, etc. Combined with MLST used to differentiate *Bacillus* species and the results of nonredundant (NR) gene annotation (Figure S1), the strain was identified as a member of the genus *Bacillus* and was designated *B. subtilis* Y4X3.

### 3.2. Analysis of Extracellular Enzyme Production

**Figure 2:**
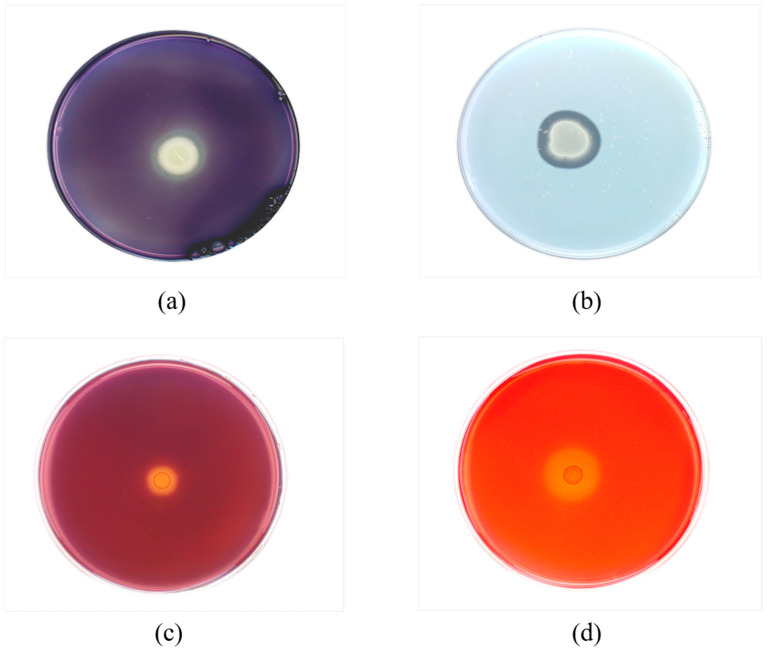
Transparent hydrolysis circles formed by *Bacillus subtilis* Y4X3 on plates. (**a**) Amylase identification medium. (**b**) Protease identification medium. (**c**) Xylanase identification medium. (**d**) Cellulase identification medium.

When the amylase identification medium was stained with 1% Lucid’s iodine solution for 1 min, a clear transparent halo appeared around the *B. subtilis* Y4X3 colony (Figure 2a), indicating that it could produce extracellular amylase. On the protease identification medium, the *B. subtilis* Y4X3 strain clearly formed transparent circles around the colonies after incubation (Figure 2b), confirming its ability to produce extracellular protease. For xylanase or cellulase identification, the medium was stained with 1 g/L Congo red solution for 10 min and rinsed with NaCl solution three times; xylanase- or cellulase-producing strains would form a transparent circle around the colony on this medium. After staining, the *B. subtilis* Y4X3 strain displayed prominent degradation circles (Figure 2c,d), indicating its ability to hydrolyze xylan and cellulose. The ratio of the diameter of the degradation circle to the diameter of the colony indicated that the *B. subtilis* Y4X3 strain had excellent cellulase production ability. The ability of the *B. subtilis* Y4X3 strain to produce extracellular enzymes reflects the evolutionary adaptations of microorganisms to their environments. This strain has potential applications in the construction and optimization of biosynthetic pathways and industrial biotechnology.

### 3.3. Genome Characterization

The whole genome of *B. subtilis* Y4X3 was sequenced, and genome assembly was performed using Unicycler software (Version: 0.5.0), integrating both second- and third-generation data. The assembled genome spans 4,215,636 bp with 43.51% G + C content, including 4438 coding genes, 100 tRNAs, and 30 rRNAs (Table 1).

**Figure 3:**
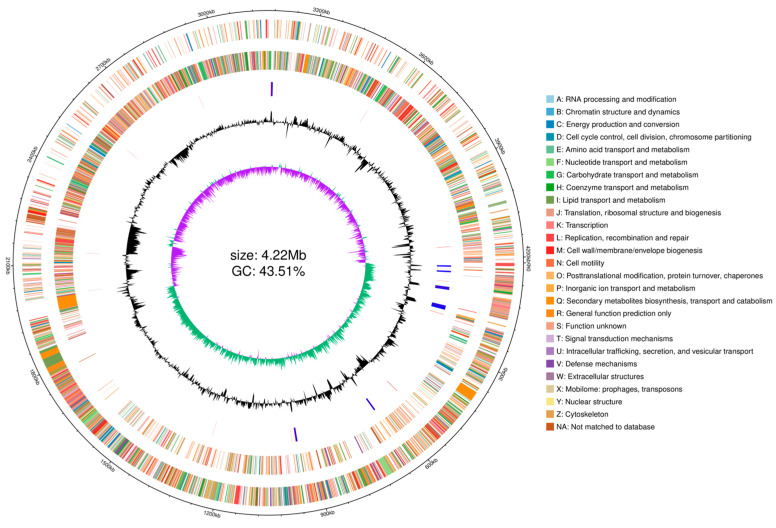
Circular genomic map of *Bacillus subtilis* Y4X3. From the outside to the inside: first circle, genomic coordinates; second circle, genes on the positive strand of the genome sequence, with different colors representing different COG functional classifications; third circle, genes on the negative strand of the genomic sequence, with different colors representing different COG functional classifications; fourth circle, rRNAs and tRNAs in the genomic sequence, with rRNAs in blue and tRNAs in red; fifth circle, GC content profile of the genomic sequence, with a sliding window of 2000 bp; sixth circle, GC skew curve of the genomic sequence, with a sliding window of 2000 bp. Green color indicates that the G content is greater than the C content, and purple color indicates that the G content is less than the C content.

The genomic information obtained by the prediction, such as genomic structure annotation information, GC distribution, and genomic COG function annotation, was integrated using the R package (Version: 0.4.11) circlize to map the genome circles (Figure 3).

### 3.4. Functional Annotation of the Genome

To gain a comprehensive understanding of gene function in the *B. subtilis* Y4X3 strain, its genome was annotated using eight databases: the UniProt, KEGG and KEGG Pathway, GO, Pfam, COG, TIGRFams, RefSeq, and NR databases. The results of the annotation are summarized in Table S1.

#### 3.4.1. COG Annotations

In the COG database, gene products are classified into direct homologs. A total of 3334 coding genes of *B. subtilis* Y4X3 were annotated in the COG database and categorized into 26 functional groups (Figure S2). Among the COG categories of metabolism, the most abundant category was ‘General function prediction only’, which contained 418 genes and accounted for 12.54% of the total genes. The categories ‘Amino acid transport and metabolism’, ‘Transcription’, and ‘Carbohydrate transport and metabolism’ contained 377, 350, and 337 genes, respectively, representing 12.54%, 11.31%, and 10.11% of the total gene count. The categories ‘Extracellular structures’ and ‘Cytoskeleton’ had the fewest number of annotated genes, with only three and six genes, respectively. Additionally, 161 genes were classified as ’Function unknown’, accounting for 4.83% of the total number of genes, which may represent undiscovered genes in *B. subtilis* Y4X3.

#### 3.4.2. KEGG Annotations

The KEGG database is the main public database used for the systematic analysis of gene product metabolic pathways and their functions. A total of 2526 coding genes of *B. subtilis* Y4X3 were annotated in the KEGG database and categorized into five main groups: ‘Cellular Processes’, ‘Environmental Information Processing’, ‘Genetic Information Processing’, ‘Metabolism’, and ‘Organismal Systems’. These genes were further divided into 23 subcategories of metabolic pathways (Figure S3). Most of the annotated genes in *B. subtilis* Y4X3 were related to ‘Metabolism’, with dominant functional categories including ‘Global and overview maps’, ‘Carbohydrate metabolism’, ‘Amino acid metabolism’, and ‘Metabolism of cofactors and vitamins’. These categories contained 753, 251, 206, and 186 annotated genes, respectively. Second, 174 and 134 annotated genes were related to ‘Membrane transport’ and ‘Signal transduction’, respectively, and were involved in environmental information processing. These findings suggest that *B. subtilis* Y4X3 has robust carbohydrate metabolism and membrane translocation capacity, which increase its potential for environmental adaptation and degradation, transformation, and utilization of complex carbohydrates.

#### 3.4.3. CAZy Annotations

The CAZy database focuses on analyses of the genomic, structural, and biochemical information of carbohydrate-active enzymes. The CAZy annotation results (Table 2) identified 52 glycoside hydrolases (GHs) genes, 39 glycosy transferases (GTs) genes, 17 polysaccharide lyases (PLs) genes, 17 carbohydrate esterases (CEs) genes, 5 auxiliary activities (AAs) genes, and 7 carbohydrate-binding modules (CBMs) genes. These results indicate that GHs and GTs are predominant in *B. subtilis* Y4X3, providing the basis for the formation, transfer, and further metabolism of monosaccharides, polysaccharides, and glycosides. The GH genes may encode bioenzymes, such as cellulases, xylanases, proteases, and amylases. These enzymes play crucial physiological roles in living organisms and can also be applied in industrial production and biotechnology, such as biofuel, paper, food, and detergents, making research on these enzymes highly valuable.

### 3.5. Heterologous Expression and Purification of Cellulases

**Figure 4:**
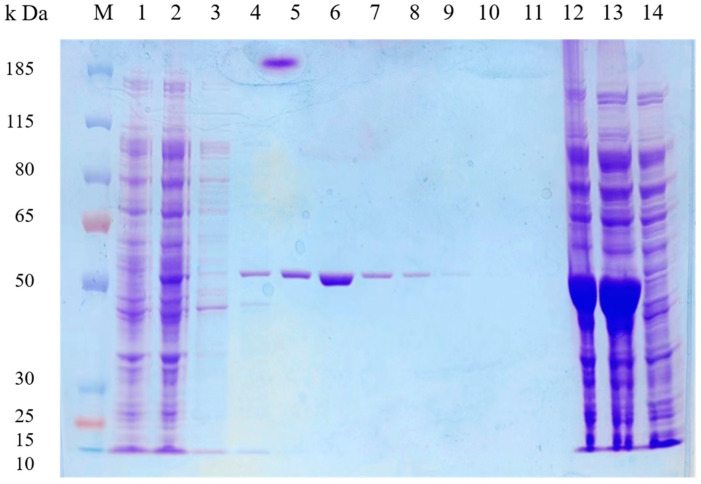
SDS-PAGE analysis of purified Cel5A. M: protein marker; lanes 1–2: pET28a strain, uninduced recombinant strain; lanes 3–11: protein elution with 0 mmol/L, 10 mmol/L, 20 mmol/L, 40 mmol/L, 60 mmol/L, 80 mmol/L, 100 mmol/L, 200 mmol/L, and 500 mmol/L imidazole solution, respectively; lanes 12–14: total protein, crude extract, and flow-through solution of Cel5A.

The *B. subtilis* Y4X3 strain was found to hydrolyze cellulose efficiently, and a cellulase (EC 3.2.1.4) gene, encoding an endoglucanase of the GH5 family, was subsequently identified through genome-wide functional annotation. The cellulase was named Cel5A for simplicity. The Cel5A gene is 1500 bp in length, encoding a protein of 499 amino acids and a predicted molecular mass of 55.3 kDa. The protein is predicted to contain a signal peptide consisting of 29 amino acids (Figure S4). Specific primers were designed to amplify the cellulase gene without the signal peptide from the genome of the *B. subtilis* Y4X3 strain. The gene was successfully transferred into *E. coli* BL21 using pET28a (+) as a vector (Figure S5). Protein expression was induced by the addition of 0.4 mM IPTG at low temperatures. Then, we used nickel column affinity chromatography, and elution with different imidazole solutions was performed to successfully obtain the purified enzyme. The size of the cellulase without the signal peptide was approximately 53.5 kDa, and the SDS-PAGE results (Figure 4) revealed that the purified enzyme formed a single band consistent with the theoretical size. The elution was most effective with a 40 mmol/L imidazole solution.

### 3.6. Biochemical Characterization of Enzymes

#### 3.6.1. Effects of pH and Temperature

**Figure 5:**
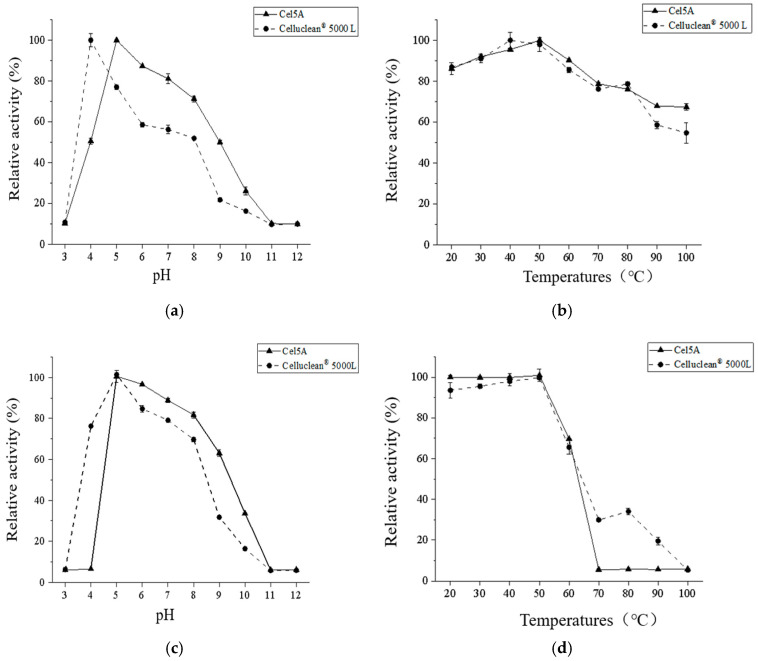
Effects of pH and temperature on enzymes: (**a**) effect of pH on enzyme activity, (**b**) effect of temperature on enzyme activity, (**c**) effect of pH on enzyme stability, (**d**) effect of temperature on enzyme stability.

The effect of pH on the CMCase activity of cellulase was determined at pH values ranging from 3.0 to 12.0 to investigate the optimum pH (Figure 5a) and pH stability (Figure 5c) of the cellulase. The purified cellulase Cel5A exhibited maximum activity in citrate-sodium citrate buffer at pH 5.0, maintaining over 50% relative enzyme activity between pH 4.0 and 9.0. The pH stability indicated that Cel5A was relatively stable in the pH range of 5.0 to 9.0, retaining more than 60% of its initial activity after 60 min of treatment. In comparison, the commercial enzyme Celluclean^®^ 5000 L has an optimum pH of 4.0 and remains stable mainly in the pH range of 4.0 to 8.0. Thus, Cel5A has a broader pH range for activity and superior stability compared with those of Celluclean^®^ 5000 L.

The optimum reaction temperature (Figure 5b) and temperature stability (Figure 5d) of cellulase were examined at temperatures ranging from 20 °C to 100 °C. The purified cellulase Cel5A was determined to have the highest enzyme activity for the catalytic reaction at 50 °C and excellent thermal stability in the range of 20–50 °C. More than approximately 70% of the initial enzyme activity was retained even after 60 min of treatment at 60 °C. However, Cel5A was almost inactivated when the temperature was increased to 70 °C, indicating high sensitivity to higher temperatures. The commercial enzyme Celluclean^®^ 5000 L has an optimum temperature of 40 °C and maintains excellent stability between 20 °C and 50 °C, which is slightly lower than the relative activity observed for Cel5A; however, Celluclean^®^ 5000 L still possesses nearly 20% or more enzyme activity at high temperatures between 70 °C and 90 °C.

Currently, Celluclean^®^ 5000 L is mainly used as a detergent ingredient in laundry detergents and gels to remove microfibers and stains caused by wear and tear. These results show that Cel5A also has the potential to be used in the laundry industry to improve washing efficiency and reduce environmental pollution due to its biodegradability.

#### 3.6.2. Substrate Specificity

To understand the substrate specificity of the cellulase Cel5A, sodium carboxymethylcellulose (CMC-Na), crystalline cellulose (Avicel), beechwood xylan, β-mannan, sucrose, and kombucha polysaccharide (laminarin) were used as hydrolyzed substrates under optimal conditions for determining enzyme activity. The results (Table 3) showed that Cel5A had the greatest hydrolytic effect on CMC-Na, with a specific enzyme activity of 13.90 U/mg; Avicel and beechwood xylan had specific enzyme activities of 3.86 U/mg and 0.97 U/mg, respectively; and no enzyme activity was detected for the other substrates. Celluclean^®^ 5000 L also showed the best degradation of CMC-Na, with a specific enzyme activity of 11.31 U/mg; it also degraded other substrates but with lower activity. These results indicate that Cel5A has substrate specificity for β-1,4 glycosidic bond-linked dextran and that its hydrolytic activity for CMC-Na is slightly more effective than that of Celluclean^®^ 5000 L.

#### 3.6.3. Effects of Metal Ions and Chemical Reagents

Enzyme activity is usually strongly affected by various inorganic and organic factors, such as detergents and cations. The enzyme activity was determined by preincubating Cel5A with different reagents at 50 °C for 30 min to assess the tolerance of Cel5A to metal ions (Table 4) and chemical reagents (Table 5). The results showed that 1 mM and 10 mM of Mn^2+^, 1 mM of Cu^2+^, 10 mM of Ca^2+^, 1 mM and 10 mM of Co^2+^, and 1 mM of Fe^3+^ significantly activated the catalytic reaction of Cel5A, whereas the other ions had minimal effects. The broad tolerance of Cel5A to a variety of metal ions enhances its potential for industrial utilization. In addition, 5 mM β-ME, 5 mM SDS, and 1% Triton X-100 had significant inhibitory effects on the enzyme activity of Cel5A; however, the effects of other chemical reagents, such as 1% Tween 20 and 1% Tween 80, had negligible effects. The tolerance of Cel5A to surfactants further supports its applicability in the detergent industry. These metal salts and chemical reagents are usually present in industrial practices and may affect enzyme activity. Therefore, the cation and chemical resistance of the cellulase Cel5A serve as an evaluation index for its industrial applications, such as in environmental waste management, functional oligosaccharide extraction, and bioethanol production.

#### 3.6.4. Kinetic Parameters

Purified cellulase was reacted with different concentrations of CMC-Na as a substrate to measure enzyme kinetic parameters (Km and Vmax). The Km and Vmax of cellulase Cel5A were 12.29 mg/mL and 23.88 U/mg, respectively. The enzyme’s affinity is strongly correlated with substrate concentration, and its activity increases with rising substrate concentration across a broad range.

## 4. Discussion

In this study, *B. subtilis* Y4X3 was successfully isolated from soil in Xinjiang, China. Its whole genome was sequenced and analyzed for genomic functions, while the cellulase produced by the strain was characterized in detail. The unique environmental conditions of the Xinjiang region provide rich resources for microbial diversity, which was further confirmed by the findings of this study. Genome analysis of the *B. subtilis* strain Y4X3 revealed its potential for cellulose degradation and utilization, which is important for the development of novel biocatalysts and biomanufacturing technologies.

The genus *Bacillus* is among the oldest and most diverse bacterial genera within the family Bacillaceae [40]. They are Gram-positive bacterium that is capable of forming endospores and grows under either aerobic or partially anaerobic conditions. Most *Bacillus* species exhibit considerable genetic and physiological diversity and are typically characterized by a C + G content of 35% to 46% and a genome size ranging from 3.35 to 5.5 Mb [41]. The *B. subtilis* Y4X3 strain, isolated in this study, with a C + G content of 43.51% and a genome size of 4.22 Mb, fits this profile. A 16S rRNA sequence analysis and comparison showed that *B. subtilis* Y4X3 shared more than 99% similarity with *B. subtilis* group sp. 2223, *B. subtilis* S35, and *B. velezensis* KLI2. The strain could be identified as belonging to the genus *Bacillus*, but the level of discrimination was limited. MLST is a phylogenetic analysis method that uses highly phylogenetically conserved core housekeeping genes for strain identification [42]. In this study, seven housekeeping genes (glpF, ilvD, pta, purH, pycA, rpoD, and tpiA) were analyzed using allelic profiling or sequence typing. Relevant data were retrieved from the PubMLST website (https://pubmlst.org/ accessed on 7 November 2023). By integrating the results of NR gene annotation and MLST, the strain was confirmed to be a member of the genus *Bacillus* and was designated *B. subtilis* Y4X3.

In terms of biotechnological and industrial applications, members of the genus *Bacillus* are considered very important industrial bacteria because of qualities such as their excellent protein secretion ability, robust growth, salinity, and heat resistance [43]. *B. subtilis* is particularly versatile [44] and has applications in enzyme production, biocontrol, probiotics, and bioremediation. These attributes make it suitable for various industrial applications, including detergents, food, biofuels, feed, pulp, and paper. For example, α-amylase produced by *B. subtilis* is highly thermostable, enabling its use in starch hydrolysis within the food and starch industries to increase the production of glucose and other valuable products [45]. Additionally, lipases from *B. subtilis* can catalyze the conversion of fats and oils into biodiesel, facilitating the production of biofuels [46]. Furthermore, *B. subtilis* can effectively utilize hydrocarbons, such as petroleum hydrocarbons, and degrade pollutants, thereby contributing to bioremediation efforts [47]. Functional analysis of the *B. subtilis* Y4X3 genome revealed numerous genes associated with amino acid transport and metabolism, transcription, carbohydrate transport and metabolism, and environmental information processing. The proteins encoded by these genes are beneficial for the adaptation of the strain to the external environment [48]. An in-depth analysis of genome function will help elucidate the molecular mechanism of strain to the external environment and provide a new pathway for efficient target protein expression. In addition to various carbohydrate metabolism-related genes annotated in the COG database or KEGG database, numerous enzymes were also annotated in the CAZy database. The dot-plate results confirmed that *B. subtilis* Y4X3 has the ability to secrete cellulase, xylanase, protease, and amylase. The above results suggest that *B. subtilis* Y4X3 is a strain that can produce a broad range of industrial enzymes and could be a potential candidate for the generation of more efficient and cost-effective industrial strains.

The cellulase Cel5A, derived from the *B. subtilis* Y4X3 strain, showed significant potential for industrial applications. The optimum reaction pH and temperature of purified Cel5A were 5.0 and 50 °C, respectively; the enzyme exhibited excellent thermal stability across a pH range of 5.0–9.0 and a temperature range of 20–60 °C. However, the pure enzyme solution may be denatured due to its sensitivity to high temperatures, and Cel5A was almost inactivated when the temperature increased to 70 °C. This phenomenon is similar to the properties of the cellulase from *Geobacillus thermoleovorans* T4 [49]. We provide a comprehensive comparison of Cel5A with other cellulases from Bacillus subtilis strains in Table 6, highlighting its wide pH and temperature range, as well as its high enzyme activity and contribution to the field of cellulase research.

Furthermore, the enzymatic properties of Cel5A were similar to those of the commercial enzyme Celluclean^®^ 5000 L, with Cel5A exhibiting a broader pH range and enhanced stability. Notably, Cel5A was tested as a purified enzyme solution, whereas Celluclean^®^ 5000 L, a commercial preparation, likely contains additives such as organic solvents, nonionic surfactants, polysaccharide aqueous solutions, and other protective agents that improve its thermal stability [55]. Celluclean^®^ 5000 L is primarily utilized as a detergent ingredient in the laundry and textile industry, suggesting that Cel5A also holds potential for applications in this sector, where it could enhance fabric finish and softness, as well as aid in the removal of impurities and stains from fibers. It is known that cellulase enzymes are commonly utilized in fruit and vegetable juice clarification processes [56] at pH values ranging from 3 to 7 and temperatures ranging from 30 °C to 50 °C; the enzymatic saccharification of lignocellulose [57,58] is also usually carried out under milder conditions, with pH values ranging from 4.8 to 5.0 and temperatures ranging from 40 °C to 50 °C. The enzymatic characteristics of Cel5A align with these conditions, indicating its potential for use in the food and beverage processing industry, as well as in biofuel production and other industrial applications. Metal ions can activate or inactivate enzymes through interactions with amino acid amines or carboxyl groups [59], and chemical reagents such as metal ion chelators (EDTA), reducing agents (β-ME), surfactants (Tween 20, Tween 80, Triton X-100), and denaturing agents (urea) may have positive or negative effects on enzyme activity [60]. Mn^2+^, Cu^2+^, Ca^2+^, Co^2+^, and Fe^3+^ significantly activated the catalytic activity of Cel5A, whereas β-ME, SDS, and Triton X-100 markedly inhibited the enzyme activity of Cel5A. This finding is consistent with the reported activation of cellulase by Mn^2+^ in *B. subtilis* CD001 [50] and Ca^2+^ and Co^2+^ in the symbiotic *B. subtilis* BC1 [53]. Kumar and Singh [61] reported the same effect of Triton X-100 and SDS on enzyme activity. The broad tolerance of Cel5A to various metal ions and chemical reagents is essential for maintaining enzyme stability and efficiency in industrial applications, allowing it to perform effectively across a wide range of conditions.

Currently, the high production cost of purified cellulases and the complexity of downstream processing remain unresolved challenges. Scaling up commercial enzyme treatment at the industrial level poses significant difficulties in maintaining reaction conditions and process economics [62]. Prabhjot Kaur et al. [63] have noted that crude enzymes can be effectively used to efficiently and economically upgrade pulp to dissolving pulp. Additionally, the use of cost-effective substrates, such as agricultural residues and industrial by-products, can significantly reduce production costs. Alternatively, optimizing fermentation parameters can enhance the reusability and stability of cellulases in industrial settings.

In summary, the genomic functional analysis of *B. subtilis* Y4X3 and characterization of its cellulase not only enhanced our understanding of the survival mechanisms of microorganisms in extreme environments but also provided novel enzyme resources and biomanufacturing strategies for industrial biotechnology. Future studies will further explore the application of these enzymes in different industrial processes and investigate methods to enhance their performance and yield through metabolic engineering.

## 5. Conclusions

The unique natural environment of Xinjiang, China, supports a diverse array of microbial resources, which may exhibit distinctive genotypes or produce metabolites with great potential industrial application value. This region serves as a valuable natural reservoir for the selection of strains that exhibit robust cellulase activity. In this study, the *B. subtilis* Y4X3 strain was isolated from saline soil in Xinjiang, China. Its whole genome was sequenced and annotated using various databases to elucidate its genomic functions. The annotation results and dot-plate results revealed that *B. subtilis* Y4X3 produces a variety of enzymes, including cellulase, protease, amylase, and xylanase. The cellulase Cel5A derived from this strain was subsequently characterized enzymatically. The analysis demonstrated that Cel5A exhibited broad pH adaptability, maintaining over 80% relative enzyme activity after one hour of treatment at pH 8.0 and over 60% relative enzyme activity at pH 9.0, indicating strong alkalinity resistance. Additionally, Cel5A exhibited excellent thermal stability at 20–60 °C, as well as robust tolerance to metal ions and chemical reagents. These suggest that Cel5A is a thermostable and alkali-resistant cellulase. Given its high activity and stability, our cellulase has the potential to significantly improve the efficiency of cellulose degradation in industrial settings. This could lead to more sustainable and economically viable processes in sectors such as bioenergy, paper manufacturing, and textile processing. The findings of this study have significant implications for the production of industrial strains and the application of cellulase in biotechnology and industrial processes.

## Figures and Tables

**Table 1:** Gene prediction results for *Bacillus subtilis* Y4X3.

Type	Number	Total_len *	Average_len *	Percentage of Genome
CDS	4438	3,850,455	868	87.95%
tRNA	100	7764	78	0.18%
23S rRNA	10	29,249	2925	0.67%
16S rRNA	10	15,470	1547	0.35%
5S rRNA	10	1110	111	0.03%
tmRNA	1	360	360	0.01%
misc_rna	100	14,384	144	0.33%

* Total_len: total length of genes obtained by prediction (bp). Average_len: predicted average gene length obtained (bp).

**Table 2:** CAZy annotation results for *Bacillus subtilis* Y4X3.

Class	Description	Number	Percentage
GHs	Glycoside Hydrolases	52	40.94%
GTs	Glycosy Transferases	39	30.71%
PLs	Polysaccharide Lyases	7	5.51%
CEs	Carbohydrate Esterases	17	13.39%
AAs	Auxiliary Activities	5	3.94%
CBMs	Carbohydrate-binding Modules	7	5.51%

**Table 3:** Substrate specificity.

Substrate (1%)	Glucan Linkage	Specific Activity (U/mg)	
		Cel5A	Celluclean^®^ 5000 L
CMC-Na	β-1,4-glucan	13.90 ± 0.57	11.31 ± 0.45
Avicel	β-1,4-glucan	3.86 ± 0.57	1.59 ± 0.22
Beechwood Xylan	β-1,4-xylan	0.97 ± 0.08	2.47 ± 0.16
β-Mannan	β-1,2/1,3-glucosamine	ND *	1.70 ± 0.07
Sucrose	α-1,2-glucan	ND	1.12 ± 0.05
Laminarin	β-1,3-glucan	ND	ND

* ND indicates that no activity was detected.

**Table 4:** Effect of metal ions on Cel5A activity.

Metal Ion	Relative Activity (%)	
	1 mM	10 mM
K^+^	98.44 ± 1.58	101.52 ± 1.86
Mn^2+^	114.33 ± 0.56	147.56 ± 0.98
Mg^2+^	96.99 ± 4.76	99.07 ± 4.94
Cu^2+^ Ca^2+^	118.60 ± 0.98 103.19 ± 0.05	93.32 ± 5.17 114.96 ± 0.71
Zn^2+^	104.90 ± 0.60	104.75 ± 0.53
Co^2+^	153.46 ± 0.18	123.68 ± 2.97
Ni^2+^	103.34 ± 0.57	102.34 ± 1.50
Fe^3+^	116.71 ± 4.26	107.91 ± 0.51
Control *	100	100

* Control: the enzyme activity in the absence of any additives was set as 100%.

**Table 5:** Effect of chemical reagents on Cel5A activity.

Chemical Reagents	Relative Activity (%)	Chemical Reagents	Relative Activity (%)
1 mM EDTA	98.16 ± 4.07	1% Tween 20	97.77 ± 0.19
5 mM EDTA	92.89 ± 6.26	1% Tween 80	103.73 ± 1.62
10 mM EDTA	96.16 ± 2.54	1% Triton X-100	91.74 ± 2.12
5 mM PMSF	93.89 ± 0.91	5% Ethanol	95.93 ± 0.00
5 mM β-ME	80.55 ± 1.42	5% Isopropanol	94.43 ± 0.00
5 mM SDS	76.40 ± 1.65	5% N-hexane	99.73 ± 0.00
5 mM Urea	102.19 ± 1.70	5% Glycerol	98.00 ± 3.76
Control *	100		

* Control: the enzyme activity in the absence of any additives was set as 100%.

**Table 6:** Comparison of enzymatic properties of cellulase Cel5A with other cellulases from Bacillus subtilis strains.

Cellulase Source	Optimal pH	Optimal Temperature	pH Stability	Temperature Stability	Enzyme Activity	Km	Vmax	Reference
*Bacillus subtilis* Y3X4	5.0	50 °C	5.0–9.0	20–60 °C	13.90 U/mg	12.29 mg/mL	23.88 U/mg	This study
*Bacillus subtilis* CD001	5.0	60 °C	3.0–6.5	50–60 °C	2.4 U/ml	0.996 mM	1.647 U/mL	[50]
*Bacillus subtilis* F3	7.0	50 °C	7.0–8.0	45–60 °C	54.20 U/mg	-	-	[51]
*Bacillus subtilis* AS3	9.2	45 °C	6.0–10.0	25–45 °C	3.33 U/mg	0.13 mg/mL	3.38 U/mg	[52]
*Bacillus subtilis* BC1	4.0	60 °C	4.0–10.0	20–60 °C	4 U/mg	1.243 mg/mL	271.3 µg/mL/min	[53]
*Bacillus subtilis* TD11	7.0	50 °C	4.0–9.0	50–70 °C	1.84 U/mg	-	-	[54]

## Data Availability

The whole-genome sequence data reported in this paper have been deposited in the Genome Warehouse in the National Genomics Data Center, Beijing Institute of Genomics, Chinese Academy of Sciences/China National Center for Bioinformation, under accession number GWHFIJW01000000 and publicly accessible at https://ngdc.cncb.ac.cn/gwh accessed on 8 January 2024. Other original contributions presented in this study were included in the article and the Supplementary Materials.

## Supplementary Materials

The following supporting information can be downloaded at: https://www.mdpi.com/article/10.3390/microorganisms13030552/s1, Figure S1: Distribution of NR species. Different colors represent different species. Figure S2: Classification of COG annotation results. Horizontal coordinates are the content of each COG classification, vertical coordinates are the number of genes. Specific functional descriptions for each COG type are shown in the legend to the right. Figure S3: Classification of KEGG Pathway results. The horizontal coordinate is the number of genes annotated under the pathway classification, the vertical coordinate is the pathway classification, and different colors represent different broad classifications. Figure S4: Signal peptide prediction of Cel5A from Bacillus subtilis Y4X3. Figure S5: PCR validation of the pET28a(+)/cel5A plasmid. M: Trans2K plus II; lanes 1-6: the pET28a(+)/ cel5A plasmid; lane 7: negative control. Table S1: statistics of coding gene annotation results.
