# Supplementary material for: Genomic Functional Analysis and Cellulase Characterization for the Enzyme-Producing Strain Bacillus subtilis Y4X3 Isolated from Saline–Alkaline Soil in Xinjiang, China
Source: Microorganisms. 2025 Feb 28;13(3):552. doi: 10.3390/microorganisms13030552 (PMC11944486; doi:10.3390/microorganisms13030552)
Supplement: Supplementary file 1 [file microorganisms-13-00552-s001.zip › microorganisms-3455938-supplementary.pdf]

Supplementary materials

Supplementary figures

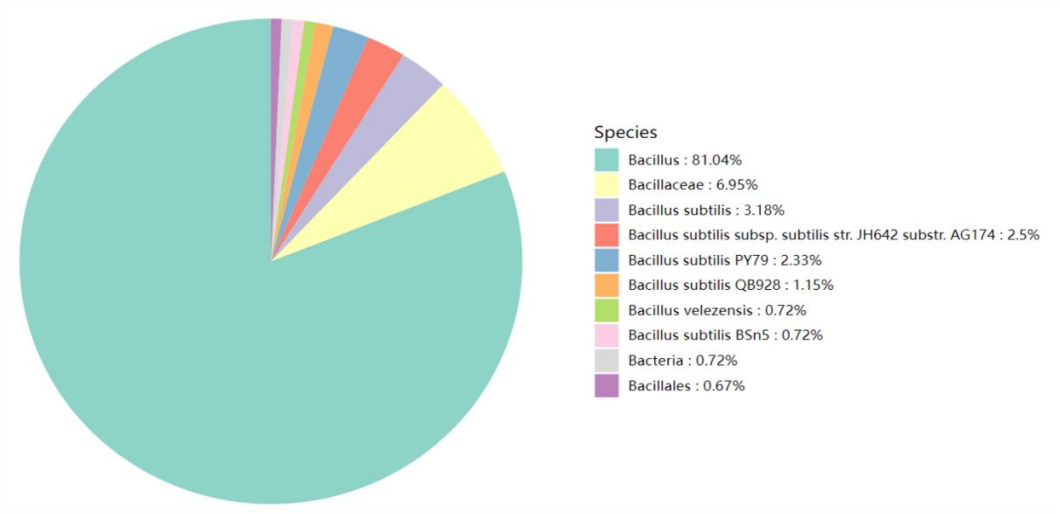

Figure S1. Distribution of NR species.Different colours represent different species.

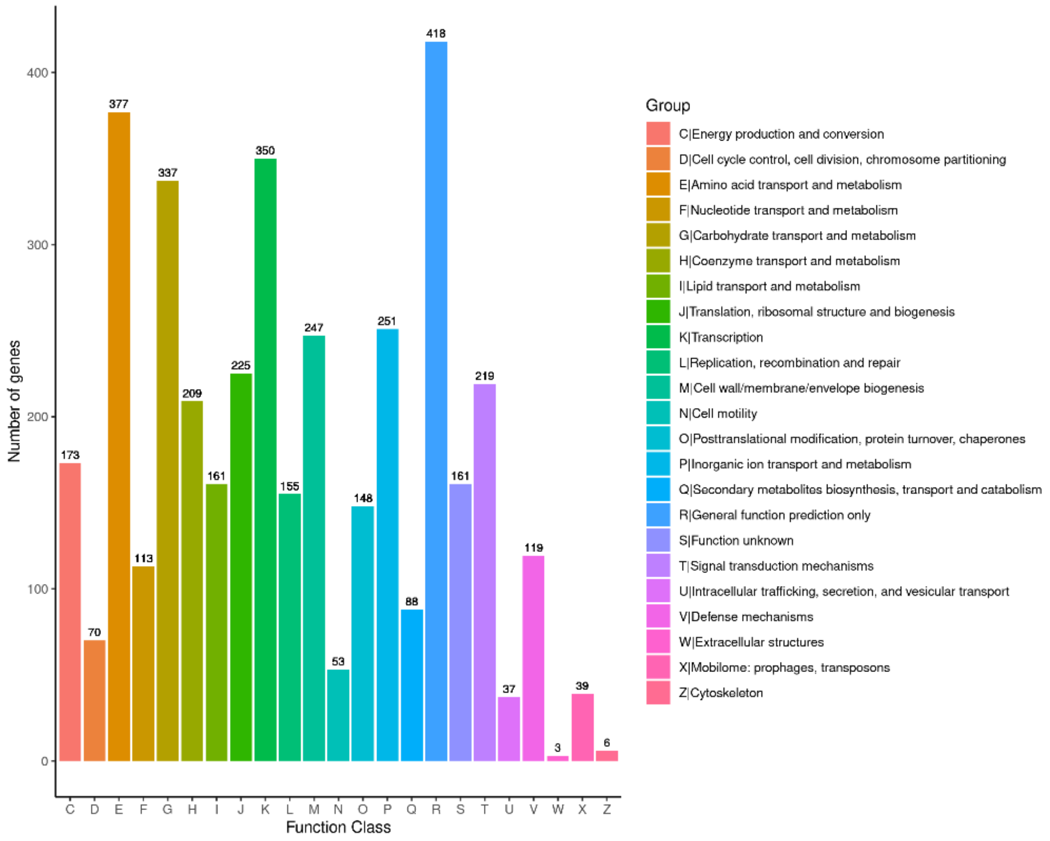

Figure S2. Classification of COG annotation results. Horizontal coordinates are the content of each COG classification, vertical coordinates are the number of genes. Specific functional descriptions for each COG type are shown in the legend to the right.

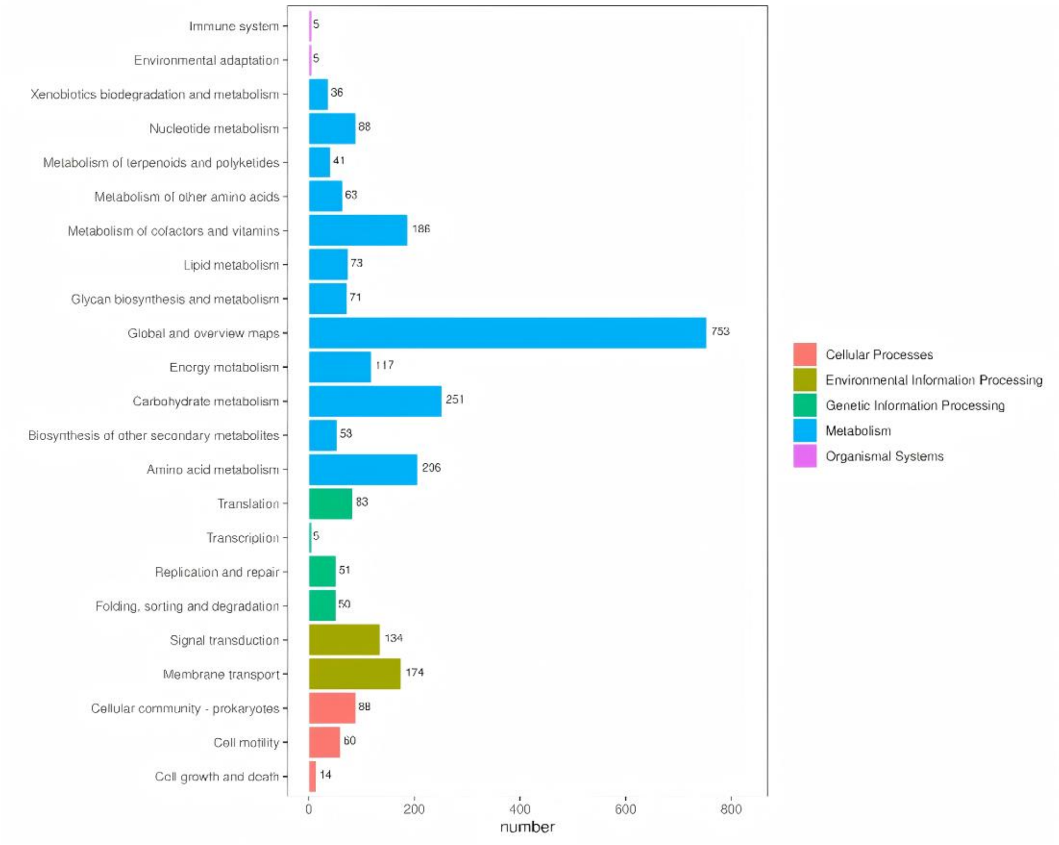

Figure S3. Classification of KEGG pathway results. The horizontal coordinate is the number of genes annotated under the Pathway classification; the vertical coordinate is the Pathway classification, and different colours represent different broad classifications.

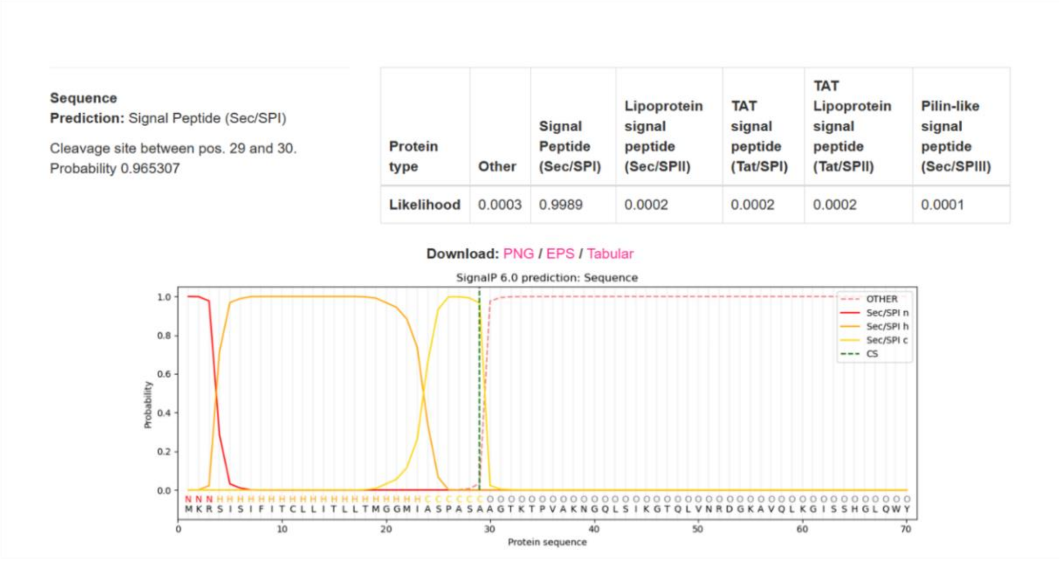

Figure S4. Signal peptide prediction of Cel5A from *Bacillus subtilis* Y4X3

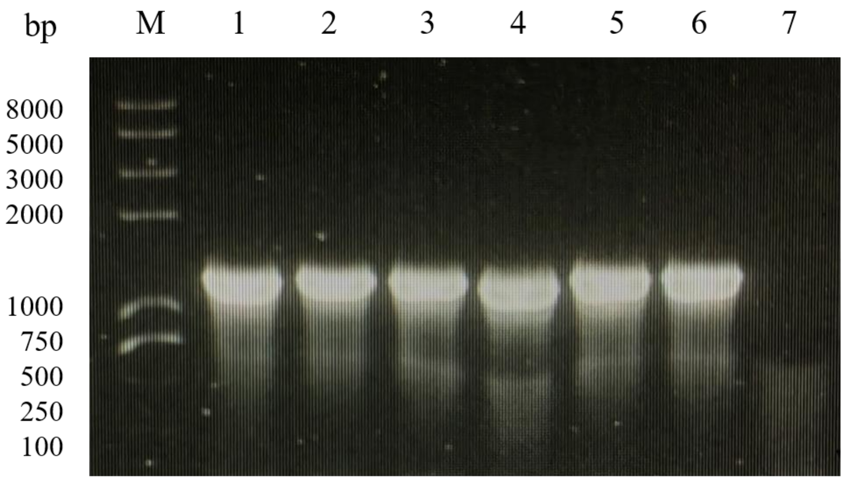

Figure S5. PCR validation of the pET28a(+)/*cel5A* plasmid. M: Trans2K plus II; lanes 1-6: the pET28a(+)/ *cel5A* plasmid; lane 7: negative control.

Supplementary Tables

Table S1. Statistics of coding gene annotation results

Item	Number	Percentage
All	4,438	100.00%
Annotation	4,391	98.94%
KEGG	2,526	56.92%
Pathway	1,310	29.52%
Nr	4,386	98.83%
Uniprot	4,364	98.33%
GO	3,338	75.21%
COG	3,334	75.12%
Pfam	3,700	83.37%
Refseq	4,358	98.20%
Tigerfam	2,328	52.46%
